# Impact de la varicocèle sur le volume testiculaire et les paramètres spermatiques

**DOI:** 10.11604/pamj.2014.19.334.4693

**Published:** 2014-11-28

**Authors:** Mohamed Hicham Benazzouz, Younes Essatara, Hachem El Sayegh, Ali Iken, Lounis Benslimane, Yassine Nouini

**Affiliations:** 1 Service d'Urologie A, Hopital Ibn Sina, CHU Rabat, Maroc

**Keywords:** Varicocèle, infertilité, volume testiculaire, paramètres spermatiques, varicocele, infertility, testicular volume, sperm parameters

## Abstract

**Introduction:**

La varicocèle est une pathologie masculine fréquente dont l'incidence est encore plus importante dans dans la population des hommes infertiles. Si ses mécanismes sont à ce jour incomplètement expliqués il semble acquis que la varicocèle peut être associée a une dysfonction testiculaire avec diminution du volume testiculaire et de la concentration en spermatozoïde de l’éjaculat.

**Méthodes:**

Dans un premier temps nous exposons les résultats d'une étude rétrospective sur 5 ans (de Mars 2009 à Mars 2014), réalisée au service d'urologie A de l'hôpital Ibn Sina de Rabat et ayant comme objectif d’évaluer l'impact de la varicocèle palpable sur le volume testiculaire et les paramètres spermatiques. Tous les patients inclus dans notre étude avaient une varicocèle palpable. Dans un deuxième temps, et à travers une revue de la littérature nous discutons l'impact du traitement de la varicocèle sur la fertilité.

**Résultats:**

39 patients ont été inclus dans notre étude. L’âge moyen était de 29,71 ans et la varicocèle siégeait dans 89,74% des cas du coté gauche. Une atrophie testiculaire homolatérale à la varicocèle était retrouvée dans 7% des cas alors que des anomalies du spermogramme se voyaient dans 69,23% des cas.

**Conclusion:**

L'impact de la varicocèle sur l'altération des paramètres spermatiques a été clairement établi bien que sa physio pathogénie ne soit pas bien élucidée. Le traitement chirurgical de la varicocèle semble indiqué chez les hommes infertiles présentant une varicocèle clinique et une altération significative du sperme.

## Introduction

La varicocèle est définit par la présence d'une dilatation variqueuse des veines du plexus pampiniforme antérieur du testicule. C'est une pathologie masculine fréquente dont l'incidence peut atteindre jusqu'a‘ 22% des hommes dans la population générale. Cette incidence est encore plus importante dans la population des hommes infertiles et est estimée à 40% lorsqu'il existe une altération du spermogramme [1]. Le mécanisme par lequel la varicocèle peut affecter la fertilité est encore à ce jour incomplètement expliqué. S'il semble acquis que la varicocèle peut être associée a une dysfonction testiculaire avec diminution du volume testiculaire et de la concentration en spermatozoïde de l’éjaculat, le retentissement du traitement de la varicocèle sur la fertilité ainsi que les options thérapeutiques restent débattus. Dans ce travail nous exposons dans un premier temps les résultats d'une étude rétrospective locale menée au service d'urologie A de l'hôpital Ibn Sina de Rabat et comparons nos résultats à ceux de la littérature. Dans un deuxième temps, et à travers une revue de la littérature, nous discutons l'impact du traitement de la varicocèle sur la fertilité.

## Méthodes

Il s'agit d'une étude rétrospective, sur une durée de 5 ans (de Mars 2009 à Mars 2014), réalisée au service d'urologie A de l'hôpital Ibn Sina de Rabat et ayant comme objectif d’évaluer l'impact de la varicocèle palpable sur le volume testiculaire et les paramètres spermatiques. Tout les patients inclus dans notre étude avaient une varicocèle palpable associée ou non à une infertilité. Les patients présentant une varicocèle infra clinique étaient systématiquement exclus.

Durant l'exploitation des dossiers cliniques, les paramètres suivant ont été étudiés: L’âge du patient; le motif de consultation; les données de l'examen clinique: les varicocèles détectées étaient classées en 3 grades: grade 1: varicocèle palpable pendant la manœuvre de Valsalva, grade 2: varicocèle palpable au repos mais non visible, grade 3: varicocèle visible et palpable au repos. L'existence d'une hypotrophie testiculaire était notée et confirmée par l’échographie; le volume testiculaire était mesuré à l’échographie selon la formule de Lambert (Longueur x largeur x hauteur x 0,71). Un volume =16ml était considéré comme normal: une échographie doppler du contenu scrotal était systématiquement demandée pour confirmer le diagnostic et préciser le grade du reflux veineux: grade 1: reflux bref (< 3 secondes) en valsalva, grade 2: reflux > 3 secondes en valsalva, grade 3: reflux permanent au repos; un spermogramme avec spermocytogramme était systématiquement demandé. Les analyses des différents paramètres spermatiques étaient basées sur la classification de l'OMS 2010 (Tableau 1) [2]. En cas de tératozoospermie, l'anomalie morphologique la plus fréquente était notée.

**Tableau 1:** Paramètres spermatiques normaux et pathologiques selon la classification de l'OMS 2010 [27]

Limite inférieure référence OMS 2010	Anomalie
Volume de sperme : 1,5 ml	<1,5ml :**hypospermie**
	>6ml :**hyperspermie**
Numération des permatozoides (SPZ) :	Absence de SPZ : **Azoospermie**
>15 millions/ml	Quelques SPZ :**Cryptozoospermie**
>39 millions/éjaculat	<15 millions/ml ou <39 millions/éjaculat :**Oligozoospermie**
Mobilité a + b 1^ère^ heure > 32%	**<32% asthénozoospermie**
Morphologie normale des SPZ :	
>15% (David modifiée)	**<**15% **:Tératozoospermie**
>4% (Kruger)	<4%
Vitalité >58%	<58% : **Nécrozoospermie**
Leucocytes < 1 million/ml	>1 million/ml : **Leucospermie**

## Résultats

39 patients présentant une varicocèle palpable ont été retenus dans notre étude.

**Figure 1:**
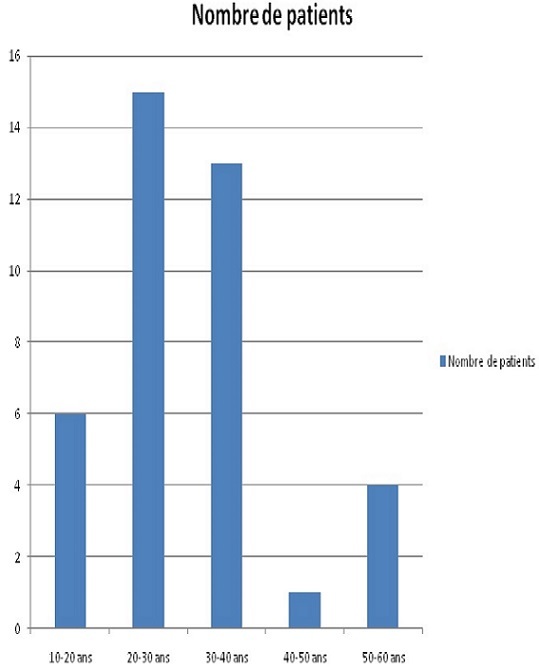
Répartition des patients en fonction de l’âge

**Age:** l’âge moyen de notre population est de 29,71 ans. La répartition des patients en fonction des tranches d’âge est représentée sur la Figure 1.

**Motif de consultation:** le motif de consultation dans notre série était principalement la douleur scrotale (31 cas soit 79,48%). Les autres motifs étaient l'autopalpation d'une masse scrotale (4 cas soit 10,26%) et l'infertilité (4 cas soit 10,26%).

**Figure 2:**
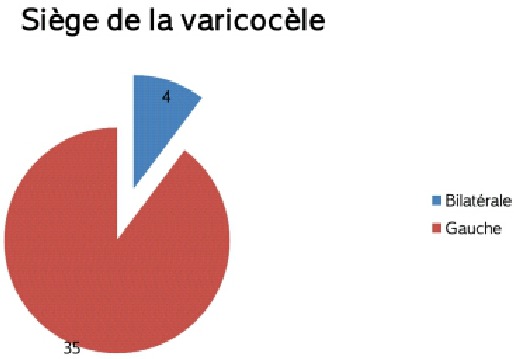
Siège de la varicocèle dans la population étudiée

**Siège de la varicocèle:** la varicocèle siégeait essentiellement du coté gauche (35 cas) alors qu'elle était bilatérale dans 4 cas. Aucun cas de varicocèle droite isolée n'a été retrouvé (Figure 2).

**Clinique:** la varicocèle palpable était le critère d'inclusion dans notre étude. Cette varicocèle était de: grade 2: dans 5 cas (11,63%); grade 3: dans 38 cas (88,37%). En ce qui concerne le volume testiculaire, 3 cas d'hypotrophie testiculaire homolatérale à la varicocèle ont été recensés.

**Données échographiques:** une échographie scrotale + doppler ainsi qu'une échographie rénale étaient réalisée chez tous nos patients.

**Le reflux veineux:** parmi les patients qui présentaient une varicocèle unilatérale, le reflux veineux était de: grade 2: dans 4 cas; grade 3: dans 31 cas. Les patients qui présentaient une varicocèle bilatérale avaient tous un reflux de grade 3 des 2 cotés.

**Le volume testiculaire:** en ce qui concerne l'impact de la varicocèle sur le volume testiculaire: une hypotrophie du testicule homolatérale à la varicocèle était retrouvée dans 3 cas (7%). Aucun cas d'hypotrophie testiculaire controlatérale n'a été noté.

**Varicocèle secondaire:** aucune tumeur rénale n'a été décelée.

**Figure 3:**
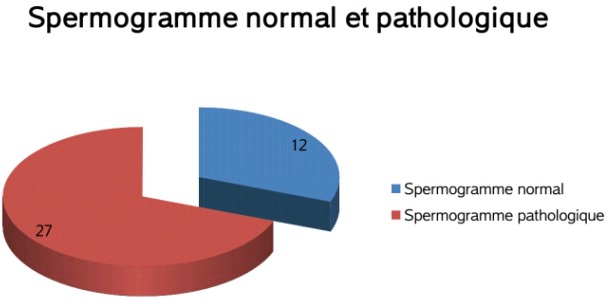
Spermogramme normal et pathologique de la population étudiée

**Paramètres spermatiques**: le Tableau 2 résume l'ensemble des paramètres spermatiques normaux et pathologiques retrouvés dans notre série. Un spermogramme normal était retrouvé chez 12 patients soit 30,77%. Alors qu'une anomalie d'au moins un des paramètres spermatiques étaient retrouvées chez 27 patients soit 69,23% (Figure 3). A noter qu'une oligo-asthéno-térato-zoospermie était retrouvée dans 8 cas (20,51%). Les anomalies morphologiques les plus fréquentes était respectivement les anomalies de la tête (microcéphalie, macrocéphalie et anomalie de l'acrosome) dans 6 cas, les anomalies de la pièce intermédiaire (reste cytoplasmiques) dans 3 cas et enfin les anomalies flagellaires (enroulement) dans un seul cas.

**Tableau 2:** Paramètres spermatiques normaux et pathologiques de la population étudiée

Paramètre spermatique	Nombre de cas et pourcentage
**Le Volume :**	
Normal :	30(77%)
Hypospermie :	8(20,5%)
Hyperspermie :	1(2,5%)
**Nombre de SPZ :**	
Normal :	17(43,67%)
Oligozoospermie :	16(41,02%)
Polyzoospermie :	3(7,69%)
Cryptozoospermie :	1(2,5%)
Azoospermie :	2(5,12%)
**Leucocytes :**	
Leucospermie :	13(33,33%)
**Mobilité des SPZ :**	
Normale :	22(56,41%)
Asthénozoospermie :	15(38,46%)
**Vitalité :**	
Normale :	23 (58,97%)
Nécrozoospermie :	14 (35,89%)
**% de formes normales :**	
Normal :	27(69,23%)
Tératozoospermie :	10(25,64%)

## Discussion

La varicocèle est définit par la présence d'une dilatation variqueuse des veines du plexus pampiniforme antérieur du testicule. Les varicocèles cliniques se développent unilatéralement à gauche dans 85% à 90% des cas. L'anatomie explique cette prédominance quasi-exclusive. Dans notre étude tout les patients avaient une varicocèle gauche et 4 d'entre eux avaient une varicocèle bilatérale.

### Atteintes spermatiques et testiculaires au cours de la varicocele

#### Atteintes testiculaires

Dans notre série une hypotrophie du testicule homolatéral à la varicocèle était retrouvée dans 7% des cas, alors qu'aucune hypotrophie controlatérale n'a été notée. Ses résultats concordent avec ceux de la World Health Organization (1992), où le volume testiculaire était fréquemment diminué du côté de la varicocèle. Deux études randomisées prospectives chez l'adolescent ont montré une augmentation de taille testiculaire homo et controlatérale après traitement de la varicocèle comparativement à ceux qui n'avaient pas été traités.

#### Atteintes spermatiques

Dans notre étude seul 30,77%des patients un spermogramme normal. Alors qu'une anomalie d'au moins un des paramètres spermatiques étaient retrouvées 69,23% des cas. Les anomalies les plus rencontrées par ordre de fréquence étaient respectivement l'oligozoospermie (41,02%), l'asthénozoospermie (38,46%), la nécrozoospermie (35,89%) et la tératozoospermie (25,64%). Le plus souvent il existait plusieurs anomalies concomitantes et l'oligo-asthéno-térato-zoospermie était retrouvée dans 20,51% des cas.

Le Tableau 3 [3] résume les anomalies les plus fréquemment retrouvées lors du spermogramme dans la littérature. Comme le décrivent F. Comhaire et A. Mahmoud [4], l'analyse du sperme retrouve le plus souvent une oligoasthénotératozoospermie. Elle s'accompagne d'un volume d’éjaculat normal, voire d'une hyperspermie, parfois d'une augmentation du nombre de cellules rondes (peroxydases négatives), marquant la libération prématurée des cellules germinales. Une augmentation de la leucospermie (cellules rondes à peroxydases positives) peut également être retrouvée. Le spermocytogramme montre classiquement des anomalies de la tête des spermatozoïdes (allongées ou amincies), des anomalies de la pièce intermédiaire à type de reste cytoplasmique (persistance de la gouttelette cytoplasmique) ou encore des anomalies flagellaires à type d'enroulement. Plus récemment, une étude de l'OMS réalisée dans 24 pays a rapporté la présence d'une varicocèle chez 25% des 3626 hommes qui avaient des anomalies spermatiques alors qu'elle n’était observée que chez 12% des 3468 hommes à sperme normal [5]. Dans cette étude, l'atteinte spermatique consistait en une altération isolée du nombre de spermatozoïdes sans atteinte de la mobilité ni de la morphologie des spermatozoïdes. En revanche, dans l’étude plus récente de Mori et al. portant sur 360 adolescents, une diminution de la mobilité et de la concentration en spermatozoïdes est associée à une varicocèle, quel que soit le grade de celle-ci [6]. Néanmoins, la diminution de la mobilité est plus importante quand le grade de la varicocèle est plus élevé. Enfin, une autre étude récente mais rétrospective menée chez 514 hommes infertiles rapporte une oligozoospermie sévère (concentration < 5 millions/mL) dans 33,7% des cas et des anomalies morphologiques comparables à celles décrites par MacLeod (microcéphalie et restes cytoplasmiques) dans 63,2% des cas [7].

**Tableau 3:** Spermogramme et spermocytogramme normaux et anomalies les plus fréquentes liées à la varicocèle [2]

Paramètres spermatiques	Valeurs seuils	Anomalies observées *
Volume éjaculé	2–6 ml	Idem ou supérieur (Grade I ou infraclinique)
Numération par éjaculat	> 40 millions	Inférieur (de oligo- à azoospermie)
Cellules rondes	< 5 millions/ml	Supérieur
Leucospermie	< 1 million / ml	Supérieur
Mobilité (a + b)	> 50 %	Inférieur
Vitalité	> 60 %	Inférieur
Pourcentage de formes normales	> 30 % (selon la classification de David)	Inférieur avec allongement de la tête, élargissement de la pièce intermédiaire et flagelles enroulés
	> 15 % (selon Kruger)	

* Ces anomalies sont variables selon les individus et peuvent s'associer entre elles

Des techniques plus récentes (Fluorescent In Situ Hybridization (FISH) - étude de la fragmentation de l'ADN) non utilisées en routine ont montré une augmentation des taux d'aneuploïdie spermatique et de la fragmentation de l'ADN spermatique [8]. Les paramètres endocriniens peuvent être altérés avec une Follicle Stimulating Hormone (FSH) élevée, inhibine B basse et testostérone subnormale. Ces altérations traduisent une atteinte sécrétoire périphérique se rencontrant dans les formes évoluées de varicocèle. Ainsi, même si la présence d'une varicocèle n'est pas synonyme d'anomalies du sperme, il apparait que la découverte d'une varicocèle est souvent associée à des altérations spermatiques sans qu'il y ait un consensus dans la littérature sur la nature et la spécificité de celles-ci.

#### Apport du traitement de la varicocele sur la fecondance naturelle

Concernant cette approche, les résultats sont contradictoires. Ainsi, la revue de The Cochrane Collaboration évoque une absence de bénéfice en termes de fécondance après traitement des varicocèles [9]. Néanmoins, il est important de signaler que la méthodologie de cette méta-analyse permettait l'inclusion de patients porteurs de varicocèles infracliniques n'ayant pas d'anomalies spermiologiques. Pour résoudre ce problème Ficarra et al [10] ont réalisé une nouvelle méta analyse à partir des mêmes études de la revue de The Cochrane Collaboration mais cette fois ci en excluant les patients avec une varicocèle infraclinique ou chez qui les paramètres spermatiques étaient normaux. Son équipe retrouvait une augmentation significative du taux de grossesses au sein des couples traités pour varicocèle (36,4%) par rapport à ceux non traités (20%).

Quant à Marmar et al. en 2007, ils indiquent un taux de grossesse de 33% chez les patients traités pour varicocèle contre 15,5% chez les non traités [11]. Cayan et al confortent ces résultats en termes de fertilité de patients traités porteurs de varicocèles palpables avec troubles du spermogramme [12]. Une grossesse spontanée postopératoire a été rapportée dans 39,07% (étude comportant 4 473 sujets). Enfin l’étude randomisée contrôlée de Abdel-meguid et al [13] retrouvait, avec un niveau de preuve 1b, une supériorité de la varicocelectomie à l'observation. 150 patients avec une infertilité de plus d'un an, une varicocèle palpable et au moins une anomalie des paramètres spermatiques étaient randomisés ( groupe traité n = 75 Vs groupe observé n = 75). Les résultats montraient un taux de grossesse spontanée significativement plus important dans le groupe traité (32.9% avec varicocelectomie vs. 13.9% avec observation, OR = 3.0.4; 95%CI = 1.33∼6.95). Ces résultats semblent confirmer le bénéfice d'une prise en charge chirurgicale ou radiologique d'une varicocèle sur les taux de grossesse spontanée.

#### Apport du traitement de la varicocele sur les paramètres spermatiques

Yamamoto et al avaient suggéré dans leur étude prospective randomisée en 1996 que le traitement des varicocèles infracliniques n'apportait pas de bénéfice en termes de fertilité [14]. Ceci a été repris et confirmé lors de recommandations plus récentes [15, 16]; les varicocèles palpables représentent la seule entité nécessitant un acte thérapeutique. Ainsi, les études évaluant l'apport du traitement de la varicocèle adoptent ce postulat et retrouvent de nettes améliorations spermiologiques [17]. En atteste la méta-analyse d'Agarwal et al, en 2007, qui soulignent une augmentation nette de la concentration du sperme variant de 9,7 millions/mL à 12 millions/mL selon la technique utilisée (ligature haute ou microchirurgie), une augmentation de la mobilité de 9,9 et 11,7% réciproquement, et une diminution de la tératospermie de 3% [18].

De façon surprenante, il a été rapporté que le traitement de la varicocèle permettrait d'obtenir la production d'une faible quantité de spermatozoïdes mobiles dans l’éjaculat des patients ayant initialement une azoospermie non obstructive due a une hypo spermatogenèse ou à un arrêt de maturation tardif [19–21]. Les conclusions de la méta-analyse réalisée en 2011 par Schlegel et Golstein colligeant 14 études ayant trait à l'intérêt d'une varicocelectomie chez des hommes présentant une azoospermie non obstructive vont dans le même sens [22]. En effet, parmi les 327 patients azoospermes ayant bénéficié d'un traitement chirurgical, 1/3 d'entre eux (119, soit 36%) ont présenté une reprise partielle de la spermatogenèse avec présence de spermatozoïdes sur au moins un éjaculat après la chirurgie. De ce fait, chez 10% de ces patients, le nombre de spermatozoïdes était suffisant pour réaliser une ICSI avec sperme éjaculé. Selon ces données, le traitement de la varicocèle pourrait ainsi permettre d’éviter la réalisation d'une biopsie testiculaire. Des améliorations en termes de fonctionnement des cellules de Leydig sont également mises en avant par Su et al en 1995 avec une augmentation de la testostéronémie.

#### Traitement de la varicocèle et reproduction médicalement assistée

Le traitement de la varicocèle ou la réalisation d'une procréation médicalement assistée sont des options pour la prise en charge d'un couple ayant une infertilité masculine et dont l'homme est porteur d'une varicocèle. Le principal argument en faveur du traitement de la varicocèle réside dans le fait de traiter une condition pathologique, susceptible de réaliser un traitement définitif de l'infertilité, à l'opposé des techniques de reproduction médicalement assistée qui doivent être répétées à chaque désir de grossesse. Les autres arguments sont: les inconnues concernant les effets à long terme de la FIV et de l'ICSI sur la descendance (effets résultants de ces techniques) et la possibilité d'un meilleur rapport coût/efficacité du traitement de la varicocèle par rapport aux techniques de PMA [23, 24]. Enfin, ne pas traiter une varicocèle peut entraîner un déclin rapide et progressif de la spermatogenèse et donc des paramètres du spermogramme risquant de compromettre la fertilité masculine ultérieure [25–27]. Le traitement de la varicocèle peut être considéré comme le traitement de première intention chez un patient ayant une oligo-asthéno-tératospermie modérée sans facteur d'infertilité féminine associé. Une FIV, avec ou sans ICSI, peut être considérée comme le traitement de première intention quand il existe un facteur d'infertilité féminine indépendant, nécessitant le recours à ces techniques. Le traitement de la varicocèle pourra être envisagé de façon concomitante pour améliorer la fécondance du sperme [21].

## Conclusion

La varicocèle est une pathologie masculine fréquente dont l'incidence est encore plus importante dans la population des hommes infertiles. Son diagnostic est essentiellement clinique. L’écho-doppler scrotal permet de quantifier le reflux veineux et d'analyser le parenchyme testiculaire. L'impact de la varicocèle sur l'altération des paramètres spermatiques a été clairement établi bien que sa physio pathogénie ne soit pas bien élucidée. Le traitement chirurgical de la varicocèle semble indiqué chez les hommes infertiles présentant une varicocèle clinique et une altération significative du sperme. En effet, la varicocelectomie ou l'embolisation pourrait être bénéfique non seulement pour obtenir une amélioration des paramètres spermatiques mais également pour prévenir leur dégradation au fil du temps. Il existe différentes modalités thérapeutiques pour réaliser la cure d'une varicocèle. Plusieurs études ont évalué ces diverses techniques tant en termes d'efficacité que de complications, mais l'hétérogénéité de celles-ci rend la comparaison difficile. C'est pourquoi les recommandations concernant le type de traitement de la varicocèle laissent libre-arbitre à l’équipe médico-chirurgicale selon son expérience.
